# Advances in Doppler optical coherence tomography and angiography

**DOI:** 10.1002/tbio.201900005

**Published:** 2019-11-20

**Authors:** Yan Li, Jason Chen, Zhongping Chen

**Affiliations:** 1 Beckman Laser Institute, University of California, Irvine, California; 2 Department of Biomedical Engineering, University of California, Irvine, California

**Keywords:** angiography, Doppler, ophthalmology, optical coherence elastography, optical coherence tomography, phase-resolved

## Abstract

Since the first demonstration of Doppler optical coherence tomography (OCT) in 1997, several functional extensions of Doppler OCT have been developed, including velocimetry, angiogram, and optical coherence elastography. These functional techniques have been widely used in research and clinical applications, particularly in ophthalmology. Here, we review the principles, representative methods, and applications of different Doppler OCT techniques, followed by discussion on the innovations, limitations, and future directions of each of these techniques.

## Graphical Abstract

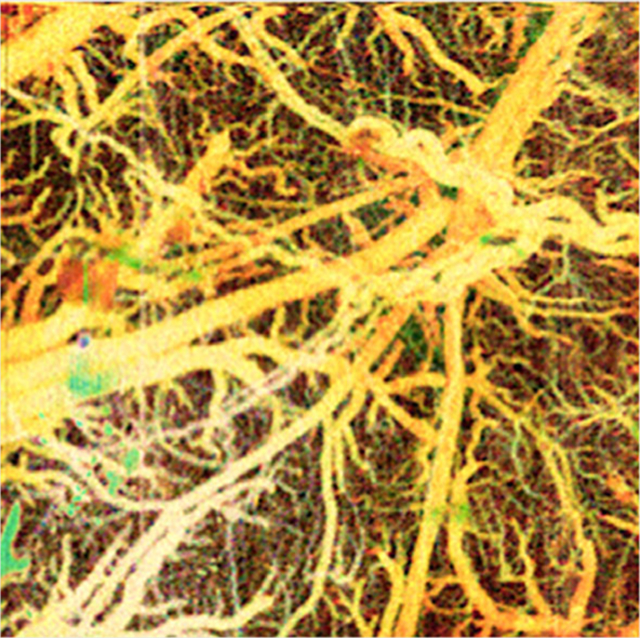

## 1 | INTRODUCTION

Optical coherence tomography (OCT) is an imaging technique that utilizes low-coherence light to capture structural images of biological tissue with high resolution in the micrometer scale [1–3]. OCT has brought great impact to diagnosis and management of diseases in many fields of medicine by enabling the visualization, hence the quantification, of morphological changes caused by the disease. However, the structural changes of the tissue are often minuscule in the earlier stages of diseases, and it is challenging to diagnose these diseases at an early stage from a morphological change with OCT imaging. Physiological changes, such as microvascular distribution and blood flow velocity, could provide complementary information in addition to OCT that may improve the diagnostic yield in early detection.

Based on the Doppler principle, Doppler OCT is a functional imaging technique that allows for quantifying the speed of moving particles with high spatial resolution and sensitivity in addition to structural imaging [4–10]. Doppler OCT was first demonstrated in 1997 [4, 5, 11] in which a spectrogram method was applied to obtain Doppler frequency shift based on time-domain OCT. However, the long acquisition time as well as the conflict between spatial resolution and velocity sensitivity limited its application [12, 13]. The development of Fourier-domain OCT significantly increased the imaging speed of OCT [14, 15]. In 2000, a phase-resolved method was proposed and demonstrated to image and quantify blood flow in which the Doppler shift could be calculated by observing the phase change between sequential A-lines in a B-scan or C-scan [6]. With the phase-resolved method, a high imaging speed, high spatial resolution and high-velocity sensitivity can be achieved. Chen et al. demonstrated the first in vivo imaging of vasculature and blood flow in patients using phase-resolved Doppler OCT [16, 17]. However, this method is sensitive to the orientation and pulsatile nature of blood flow, and the results are the most optimal when the flow direction is aligned with the probe beam. The phase-resolved Doppler variance method was developed in 2000 to address this issue, which allows quantification of transverse flow [18, 19]. In addition, Doppler variance methods also enable visualizing small vessels down to the capillary level, making it ideal for OCT angiography (OCTA) applications [18, 20]. Based on the Doppler variance method, a number of extensions have been developed, including intensity-based Doppler variance, amplitude decorrelation, speckle variance, standard deviation (SD), intensity-based differentiation, phase variance and intensity and phase-based differentiation [6–8, 10, 20–34].

OCTA has the capability to visualize the microvasculature with high resolution (1–15 μm) and a moderate penetration depth (1–2 mm). It has become an attractive tool for angiography in ophthalmology, cancer and cerebral research due to advantages over conventional imaging methods: fast acquisition time, high spatial resolution, depth-resolved information, absolute flow measurement and non-invasiveness. The qualitative comparison with the current angiography modalities is summarized in Table 1. In addition to angiography and flowmetry, Doppler OCT has also been extended to elastography application: namely, optical coherence elastography (OCE). Benefiting from the optical resolution enabled by phase-resolved OCT, OCE provides high spatial resolution at the micrometer-level and an axial displacement sensitivity on the order of subnanometer [35–40]. As such, it has been widely applied in biomedical research to provide quantitative assessment of tissue biomechanical properties [41–51].

Doppler OCT and OCTA provide a noninvasive means for studying flowmetry, angiography and elastography with high spatial resolution and sensitivity and have been utilized in the fields of neurology, ophthalmology, cardiology and dermatology [8, 16, 18, 40–42, 44, 46–50, 59–61]. In this review, we describe the methods, key advances, limitations, clinical applications and future directions of Doppler OCT.

## 2 | PRINCIPLE OF DOPPLER OCT AND ANGIOGRAPHY

### 2.1 | Spectrogram

**FIGURE 1:**
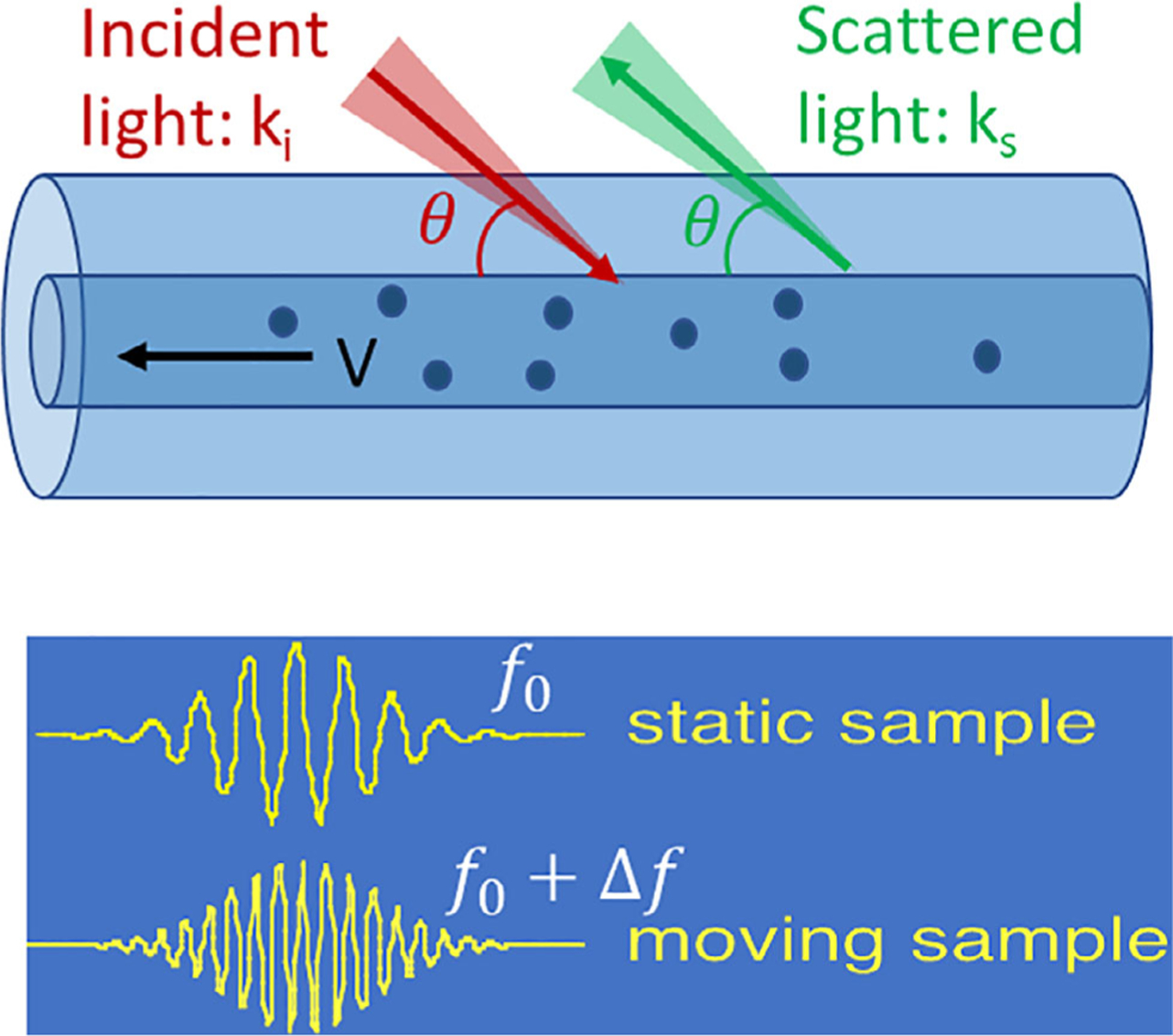
The principle of Doppler optical coherence tomography (OCT): *k*_*i*_ and *k*_*s*_ are wave vectors of incoming and scattered light, respectively. *V* is the velocity vector of the moving particles; *f*_0_: center frequency of signal from static sample; Δ*f*: Doppler frequency shift caused by moving particles. *θ* is the angle between the incident light and flow direction. Since OCT detects only back-scattered light, *θ* is identical for both the incident and back-scattered light

**FIGURE 2:**
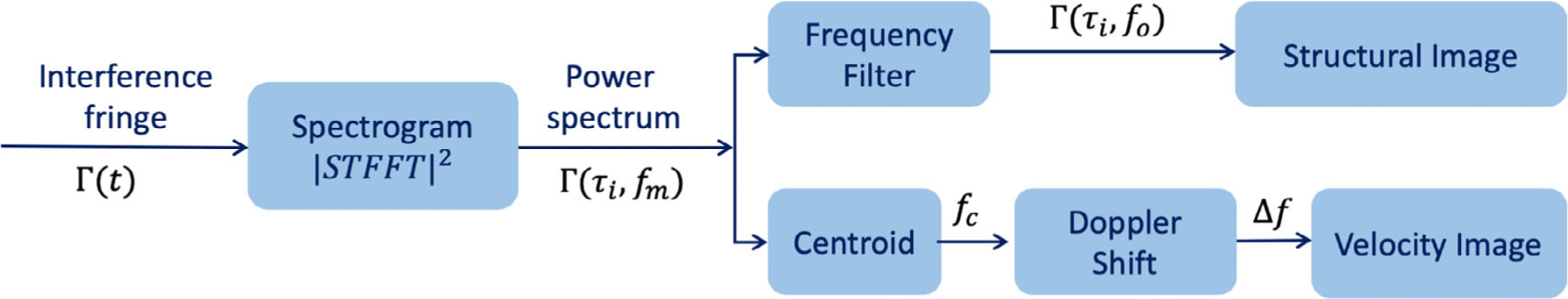
Signal processing for structural and velocity images

Doppler OCT was first proposed for blood flow quantification in 1997 [4, 5]. Based on the Doppler principle, the frequency shift of the backscattered light from a moving particle can be generated, as shown in Figure 1, and calculated using Equation (1) [4, 6]:

(1) $$\Delta f=\frac{1}{2\pi }\left(k_{\text{s}}-k_{i}\right)\cdot V$$

where ***k***_***i***_ and ***k***_***s***_ are wave vectors of incoming and scattered light, respectively, and ***V*** is the velocity vector of the moving particles. Given the Doppler angle, *θ* (between the incident light beam and the flow direction), Equation (1) is simplified to:

(2) $$\Delta f=\frac{2\times n\times V\times \text{cos}(\theta )}{\lambda }$$

where *n* is the tissue refractive index, and *λ* is the central wavelength of the light. In order to extract Doppler frequency shift, *Δf*, which is the difference between the carrier frequency from optical phase modulation, *f*_0_, and the centroid, *f*_*c*_, of the measured power spectrum, a short-time fast Fourier transformation (STFFT) or wavelet transformation can be applied to calculate the power spectrum of OCT signals [4]. A detailed signal processing is shown in Figure 2. With the spectrogram method, structural and velocity images can be obtained simultaneously, but the velocity sensitivity will be compromised by the increased spatial resolution or imaging speed.

### 2.2 | Phase-resolved Doppler OCT

To overcome the limitation of the spectrogram method, the phase-resolved Doppler OCT method was proposed in 2000 [6]. The Doppler frequency shift can be extracted by calculating the phase change in sequential A-lines using inter-A-lines or inter-frames. Deriving *Δf* through phase change can be achieved through Equation (3):

(3) $$\Delta f=\frac{\Delta \varphi }{2\times \pi \times \Delta T}$$

where *ΔT* is the time interval between sequential A-lines, and Δφ is the phase change. Δφ can be calculated using OCT complex data (*F*_*m*_ and *F*_*m* + 1_), as shown in Equation (4).

(4) $$\Delta \varphi ={\text{tan}}^{-1}\left[\frac{Im\left(F_{m}\times F_{m+1}^{*}\right)}{\text{Re}\left(F_{m}\times F_{m+1}^{*}\right)}\right]$$

where *F*_*m*_ and *F*_*m* + 1_ are the OCT complex data from same location but at a different time. Therefore, the longitudinal flow velocity can be determined by measuring the phase of the OCT signals as a function of time, as demonstrated by combining Equations (2), (3) and (4):

(5) $$V\times \text{cos}(\theta )=\frac{\lambda \times \Delta \varphi }{4\times \pi \times n\times \Delta T}=\frac{\lambda }{4\times \pi \times n\times \Delta T}\;\times {\text{tan}}^{-1}\left[\frac{Im\left(F_{m}\times F_{m+1}^{*}\right)}{\text{Re}\left(F_{m}\times F_{m+1}^{*}\right)}\right]$$

**FIGURE 3:**
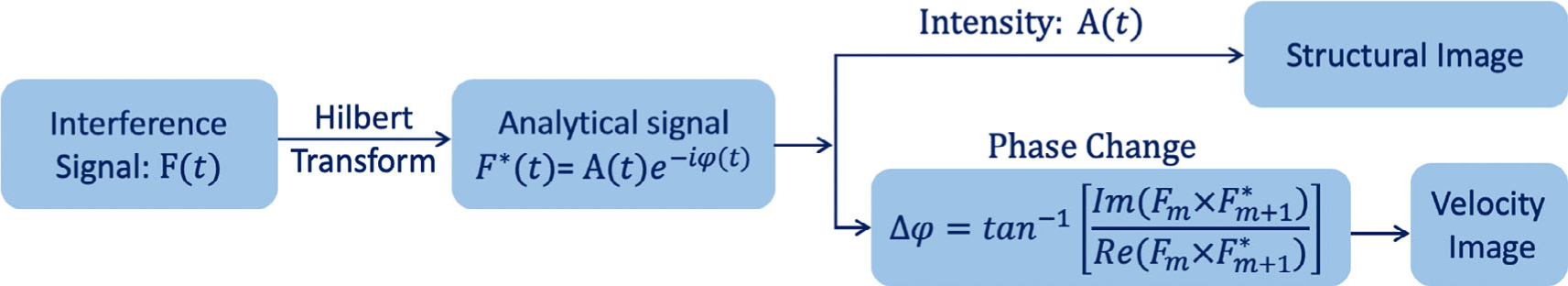
Phase-resolved Doppler optical coherence tomography method

The signal processing of phase-resolved Doppler is shown in Figure 3 [62, 63]. With the phase-resolved Doppler OCT method, high-velocity sensitivity, high spatial resolution and high imaging speed can be achieved simultaneously, enabling real-time visualization and quantification of blood flow. Since the phase-resolved Doppler OCT method is sensitive to the orientation and pulsatile nature of blood flow, determining the Doppler angle plays an important role in accurate quantification of blood flow. Furthermore, this method cannot be applied when the Doppler angle is near 90°, which limits its application, such as for ocular blood flow imaging.

### 2.3 | Doppler variance OCT

**FIGURE 4:**
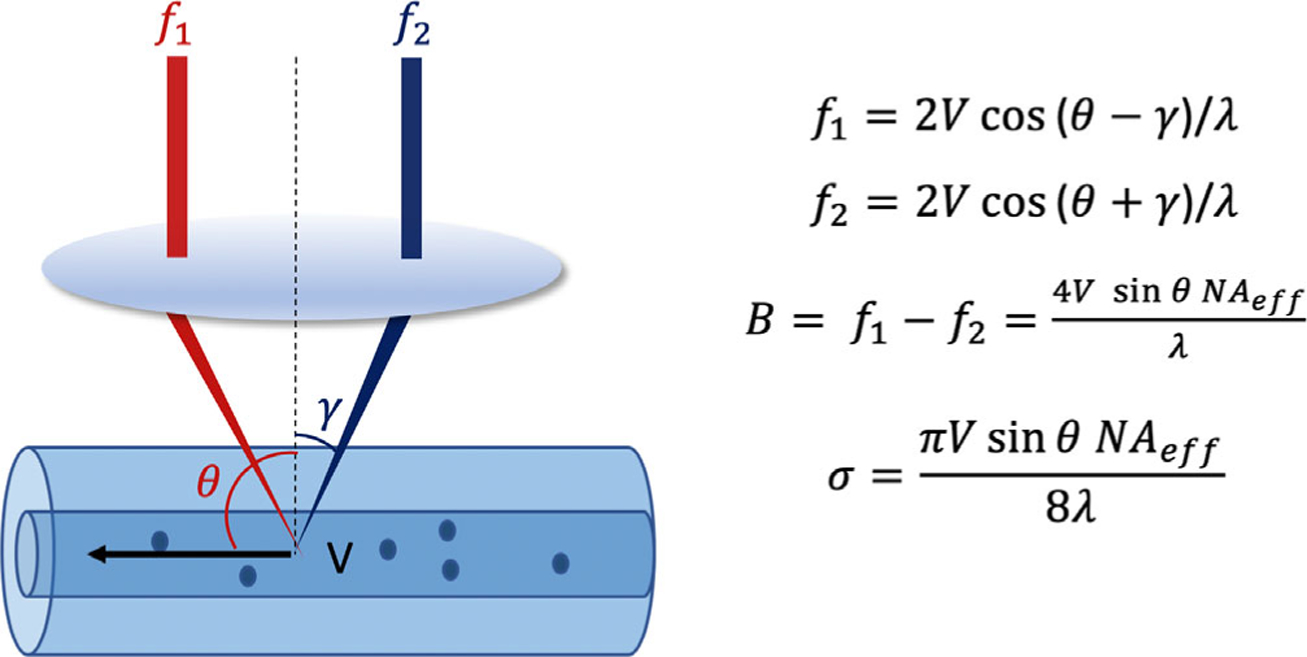
Phase-resolved variance Doppler optical coherence tomography

To address the limitations of phase-resolved Doppler OCT to image transverse flow, Doppler variance method based on the bandwidth of Doppler frequency shift was proposed [18, 19]. OCT incident probe-beam geometry causes a broadening of the Doppler frequency shift spectrum which can be used to quantify blood flow when the flow direction is near perpendicular to the probe beam. The principle is shown in Figure 4, where Doppler bandwidth, B, is approximated by the differences between the Doppler shift generated by the red and blue beam.

**FIGURE 5:**
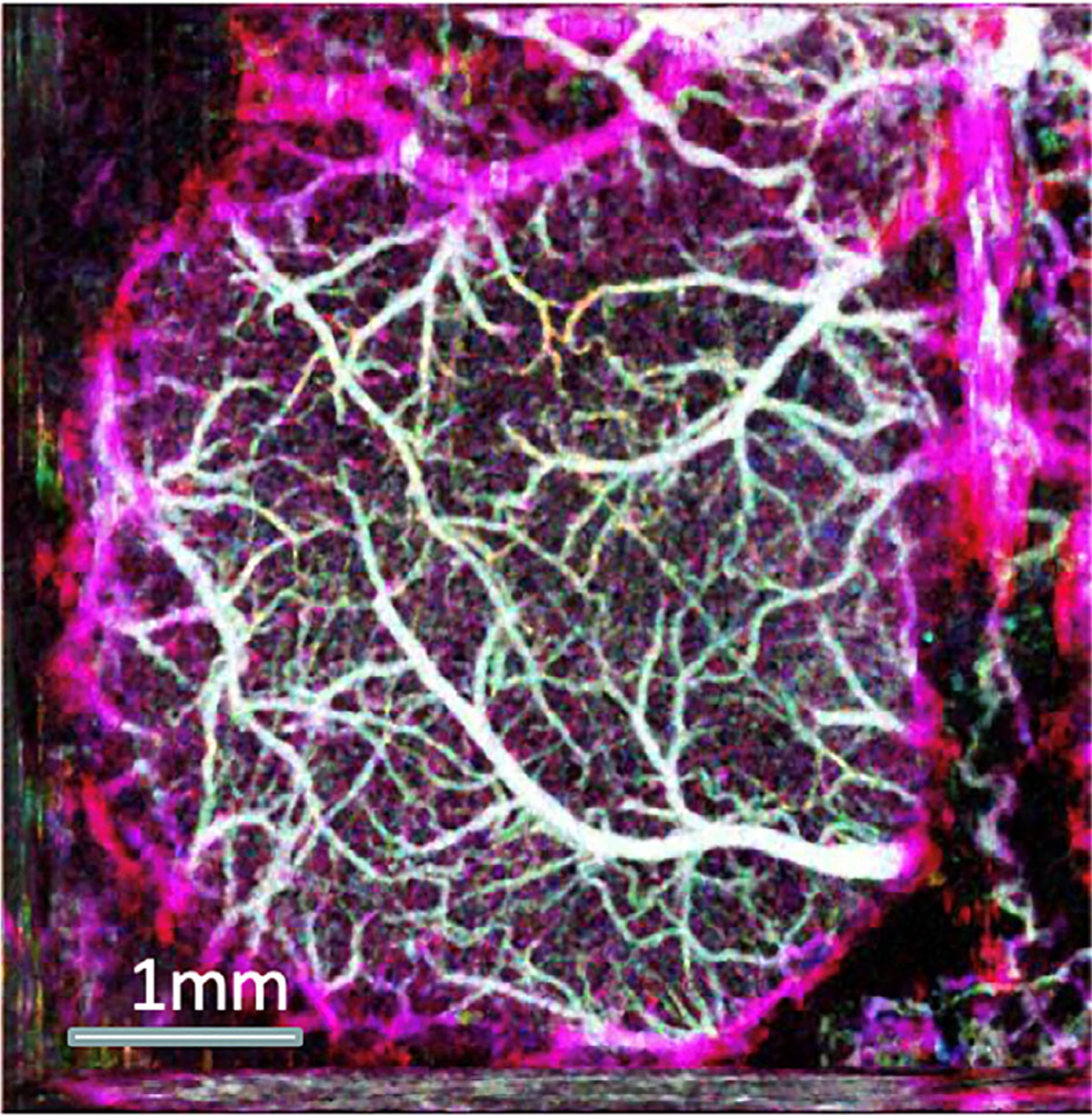
Depth-encoded image of microvasculature of rat cerebral cortex. Adapted from Reference [64]

Therefore, the transverse flow velocity, *V*_*T*_ = *V*sin*θ*, can be quantified by Equation (6) [19]:

(6) $$V\times \text{sin}(\theta )=\frac{8\times \lambda \times \sigma }{\pi \times n\times {\text{NA}}_{eff}}$$

where NA_*eff*_ is the effective numerical aperture of the scan lens. The SD, *σ*, of the Doppler bandwidth can be determined by:

(7) $$\sigma^{2}=\frac{\int g(f){\left(f-\bar{f}\right)}^{2}df}{\int g(f)f^{2}df}=\frac{1}{{\left(2\pi \Delta T\right)}^{2}}\left(1-\frac{2\times \left|F_{m}\times F_{\left(m+1\right)}^{*}\right|}{{\left|F_{m}\right|}^{2}+{\left|F_{\left|m+1\right|}\right|}^{2}}\right)$$

where *f* is the Doppler shift, $\bar{f}$ is averaged Doppler shift, and *g*(*f*) is the Doppler power spectrum. Figure 5 shows a representative angiogram from a rat cerebral cortex [8].

While Equations (5), (6), and (7) provide the back-bone for high-sensitivity flow measurement, the velocity range is limited due to phase wrapping and phase washout, which are the main challenges of Doppler OCT in flow velocity quantification. To address this, several phase calculation algorithms, such as the fast phase unwrapping method proposed by Schofield et al. have been developed [9, 65, 66].

### 2.4 | Angiogram

OCTA is an extension of Doppler OCT that reconstructs the microvasculature by detecting the micro motions in biological tissue. These motions induced by the moving blood cells and plasma can generate fluctuations in the amplitude and phase of the interference signal that correspond to the flow velocity. The first OCTA based on Doppler variance OCT was demonstrated in 2001 [17, 18], and since then various OCTA algorithms based on the detection of fluctuations in the amplitude and/or phase have been developed for the visualization of blood vessels. OCTA can be categorized into: (a) amplitude, including intensity-based Doppler variance, amplitude decorrelation, speckle variance, SD and intensity-based differentiation; (b) phase, including phase variance; and (c) both amplitude and phase, including phase-resolved Doppler variance, and intensity and phase-based differentiation. These algorithms are summarized in Table 2.

**FIGURE 6:**
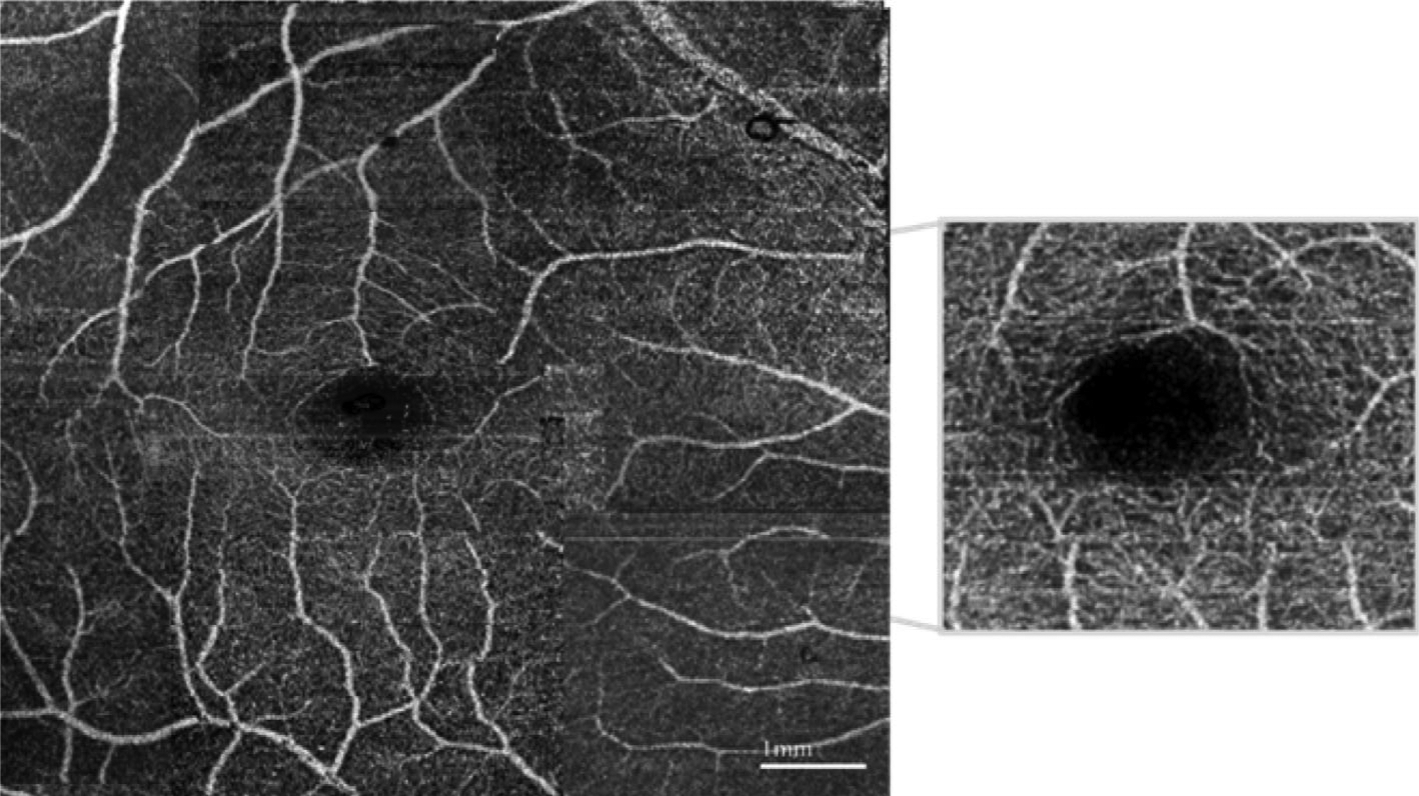
Retinal angiogram using intensity-based Doppler variance. Adapted from Reference [68]

In most cases, these algorithms have similar performances, although each is designed to utilize a particular scanning protocol for a specific application in a subfield of medicine, whose requirements vastly differ. In ophthalmology, for instance, the phase variance method is a more favorable approach for achieving a higher contrast-to-noise ratio than the amplitude decorrelation and speckle variance approaches [25], whereas in mouse brain imaging, intensity-based Doppler variance is a more suitable technique for mapping vasculature than phase-resolved Doppler variance [67]. Figure 6 shows a representative retinal angiogram (scan area: ~7 × 7 mm^2^) using intensity-based Doppler variance [68]. Microvascular network from millimeter-vessel down to single capillary can be clearly visualized.

## 3 | IMAGING PROTOCOL

OCTA acquires multiple images in sequence to reveal the portion with fluctuations. Since this principle involves temporal imaging, the imaging protocol which determines the time interval between successive fluctuations plays a key factor in the signal-to-noise ratio (SNR) and dynamic range of OCTA. The two conventional imaging protocols are inter-frames and inter-A-lines, as depicted in Table 3. In the inter-frame imaging protocol, neighboring B-scans are compared to extract vascular information. This protocol has a longer time interval Δ*T* as it utilizes the slow scan of the scanning apparatus. While this provides high sensitivity for the blood vessel with slow flow, prolonged time intervals may cause more motion-induced artifacts and phase wrapping, as well as signal saturation for the blood vessel with fast flow. On the contrary, neighboring A-lines are correlated in the inter-A-line method by using the fast scan of the scan setup to achieve a shorter time interval, and this allows for accurate quantification of fast flow while sacrificing the sensitivity for capillaries. For both imaging protocols, the scanning step needs to be much smaller with respect to lateral resolution (ie, the beam size) in order to achieve accurate angiography.

Several averaging methods have also been incorporated in imaging protocols to enhance the sensitivity of OCTA, with split spectrum and volume averaging most predominantly used, as shown in Table 4. The split spectrum method divides the interference spectrum into several narrow spectra using a Gaussian window to generate several OCT images by performing Fourier transform for each sub-spectrum [21]. These OCT images are post-processed using an angiography algorithm and then averaged to improve the SNR. This method is computationally inexpensive but sacrifices spatial resolution. The split spectrum method improves the image contrast and continuity of vessels [21]. Conversely, volumetric averaging maintains the image spatial resolution and, therefore, can greatly improve the image quality, but it reduces the imaging speed. Nonetheless, volumetric averaging is particularly advantageous in visualizing the outer capillary plexus [25].

## 4 | APPLICATIONS

### 4.1 | Ophthalmology

OCT in ophthalmology is currently most well-adapted for clinical application. To date, many OCT devices with angiography are commercially available, including ZEISS Angioplex, Optovue AngioVue, Topcon, etc. These devices aid in visualizing the vascular anatomy to allow for better understanding of the pathophysiology of eye disease. The density, morphology and flow velocity of the vasculature in the retina are highly associated with disease pathology and being able to provide quantitative measurements of these parameters can therefore provide information for early detection, disease progression monitoring and treatment management. As such, OCTA is widely used in clinical research for characterizing various eye diseases, including: (a) dry age-related macular degeneration (AMD) where choriocapillaris flow and density are associated with the disease progression [69]; (b) wet AMD which is characterized by the presence of choroidal neovascularization [23]; (c) diabetic retinopathy which exhibits abnormalities in choriocapillaris and/or retinal microvascular network [24]; (d) retinal artery/vein occlusion in which non-perfusion in the capillary can be visualized; (e) glaucoma, which can be identified by an attenuated dense peripapillary microvascular network in both the superficial disc vasculature and the deeper lamina cribosa; (f) anterior segment ischemia (ASI) where iris vessel filling function and qualitative vessel density values can be evaluated to determine whether a patient is at risk to develop ASI during strabismus surgery [70, 71]; and (g) ocular surface disorders where conjunctival and intrascleral vasculatures can be imaged for quantitative analysis of vessel density, vessel length density, vessel diameter index and fractal dimension of superficial- and deep-layer flows [72]. Currently, techniques, such as Hessian filtering, adaptive thresholding, variable interscan time analysis, machine learning and other numerical methods, have been utilized to quantify density, morphology, and flow velocity of the vasculature of the eye globe as well as suspicious lesion segmentation [73–77].

**FIGURE 7:**
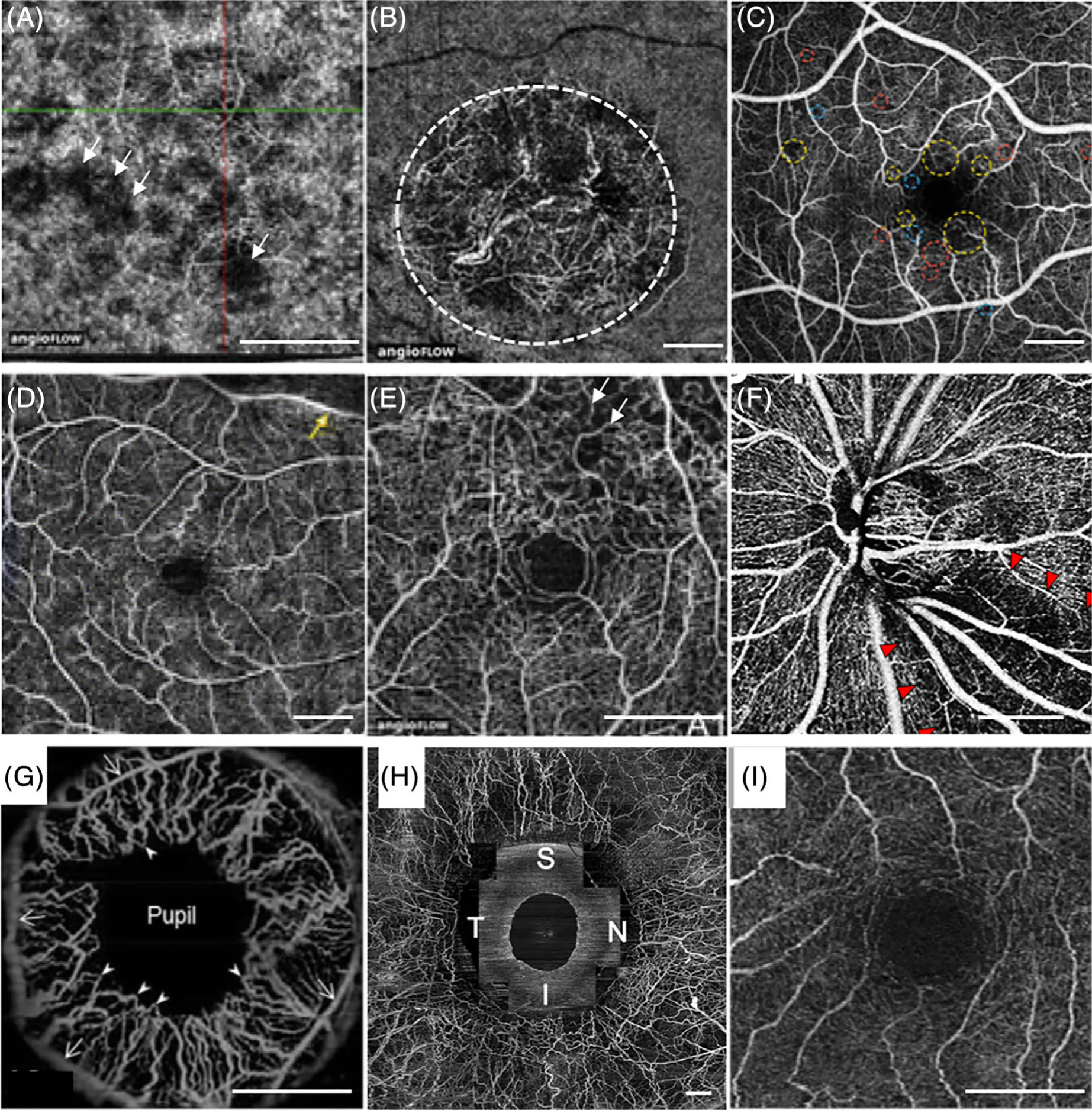
(A) Dry AMD: decreased signal in the choriocapillaris corresponding to flow impairment, indicated by the white arrows. (B) Wet AMD: presence of abnormal blood vessels, indicated by the white dashed circle. (C) Diabetic retinopathy: enlarged foveal avascular zone and aneurysms, in which microaneurysms with non-proliferative diabetic retinopathy are labeled by red dashed circles. (D) Branch retinal artery occlusion: decreased capillary perfusion, indicated by the yellow arrow. (E) Chronic branch retinal vein occlusion: capillary non-perfusion, indicated by the white arrows. (F) Glaucoma: vascular impairments, indicated by the red arrows. (G) Iris vascular network. (H) Conjunctival and intrascleral vasculatures. (I) Retinal vascular from a patient with Alzheimer’s Disease, which has a lesser vascular density. Scale bars: 1 mm. Adapted from References [24, 26, 72, 78, 79]

Figure 7 shows the representative OCTA images of the aforementioned diseases, where degradation of microvasculature can be clearly visualized. Furthermore, it has also been demonstrated that the retinal vascular density is significantly lower in Alzheimer’s patients than healthy subjects, verifying the potential of OCTA in studying Alzheimer’s disease (AD) progression through quantification of retinal vasculature change correlated to neurodegeneration [78].

### 4.2 | Neurology

**FIGURE 8:**
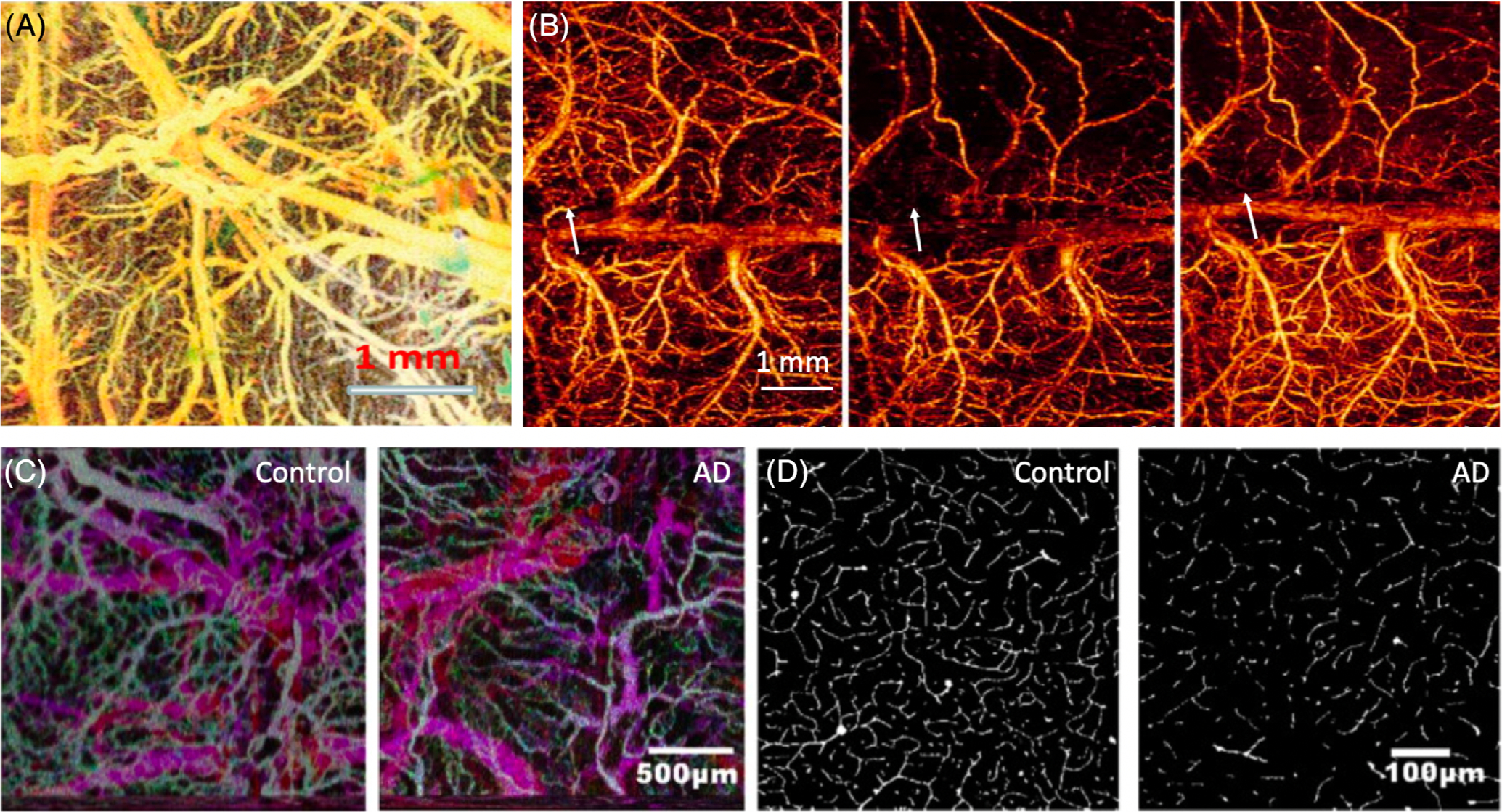
(A) The projection of vasculature of a rat cerebral cortex with thinned skull. The colormap is depth encoded. (B) The projection of vasculature from mouse brain in ischemic stroke. Left: baseline; middle: stroke; right: after 30 minutes. Blood perfusion restored partially, and occlusion still exists. The colormap is flow velocity encoded. (C) The vasculature projection of mouse brains from an AD model and healthy mouse. The colormap is depth encoded. (D)Images of vessel density differences between a control and an AD mouse, in which the AD mouse exhibited a vessel volume fraction decrease of 29% compared to the control mouse. Adapted from References [8, 60, 80]

The nervous system is a complex network which is supplied with oxygen and nutrients through the blood vessel system to maintain physiological functions. Visualizing microvasculature and quantifying blood flow velocity using OCTA play an important role in studying physiological functions of the neuron system, including occurrences and progression of brain diseases, drug administration and responses of brain to external stimuli. Due to the limited penetration depth of OCT, most current research focuses on small animal models to study the mechanism of brain injury, disease progression, and evaluation of treatment strategies. Chen et al. demonstrated the first Doppler OCT image of brain microvasculature in 1999 [12]. Liu et al. demonstrated the microvasculature from a healthy rat cortex with thinned skull, as shown in Figure 8A [8]. Jia et al. studied cerebrovascular blood perfusion in a cerebral stroke rodent model using OCTA to better understand stroke as well as to optimize current therapies via treatment monitoring [60], as shown in Figure 8B. To study traumatic brain injury (TBI), Jia et al reconstructed three-dimensional images of cerebral vasculatures in a TBI mouse model, demonstrating the microvasculature change in pre- and post-TBI mice that allows for exploring the mechanism of TBI rehabilitation [61]. Lin et al imaged the mouse brain from a 20-month 3xTg-AD model mouse to investigate the relationship between amyloid-β and vascular pathophysiology in which 3xTg-AD mice exhibited a vessel volume fraction decrease of 29% compared to the control mouse [80], as shown in Figures 8C and D.

### 4.3 | Cancer angiogenesis

**FIGURE 9:**
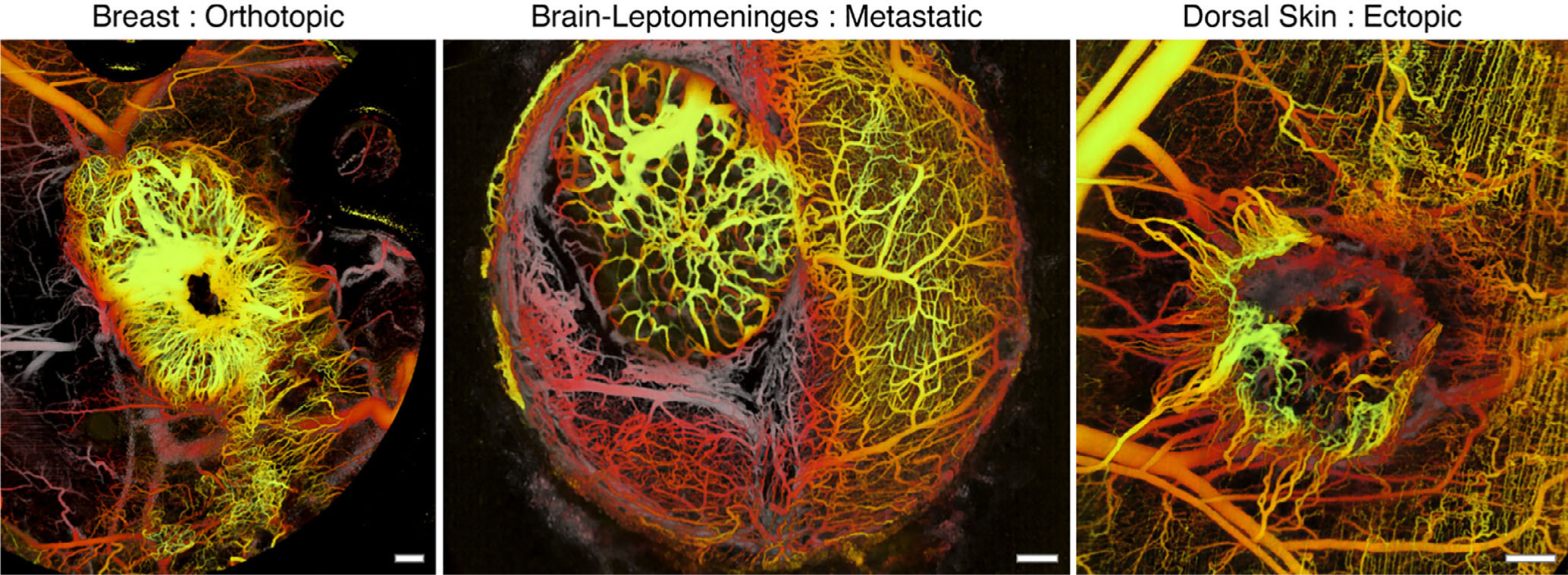
Vasculature of murine mammary carcinoma in breast (left), brain (middle), and dorsal skin (right) in which tissue microenvironments exhibit strikingly different vascular networks. Scale bar: 500 μm. The colormap is depth encoded. Adapted from Reference [82]

Tumor growth and metastasis rely on angiogenesis to provide a sufficient supply of oxygen and nutrients as well as to remove the waste [81]. Microvasculature visualization and blood flow quantification allow for early detection, characterization and treatment optimization. Vakoc et al utilized OCTA to demonstrate microvasculature of different kinds of tumors, including in the breast, brain, colorectum, and skin, as shown in Figures 9A–C [82]. The tumors can be identified by the increase in vascular density. In addition, Vakoc et al. investigated the vascular dynamics based on dorsal tumor of a rat during anti-angiogenic therapy to further verify the capability of OCTA in which a decreased mean vessel diameter as well as a continuous expanding volume of tumor were demonstrated. Alison et al. reported a study in the investigation of tumor vasculature change in malignant iris melanomas and benign iris lesions [83]. Osman et al. developed an ultrahigh-speed endoscopic OCTA system for delineation of dysplastic margins at the gastroesophageal junction and demonstrated a sensitivity of 94% and a specificity of 69% for identifying dysplasia based on 32 patients [84]. Lee et al. reported the use of endoscopic OCTA to differentiate low-grade and high-grade dysplasia in Barrett’s esophagus from 32 patients [85].

### 4.4 | Cilia motion

**FIGURE 10:**
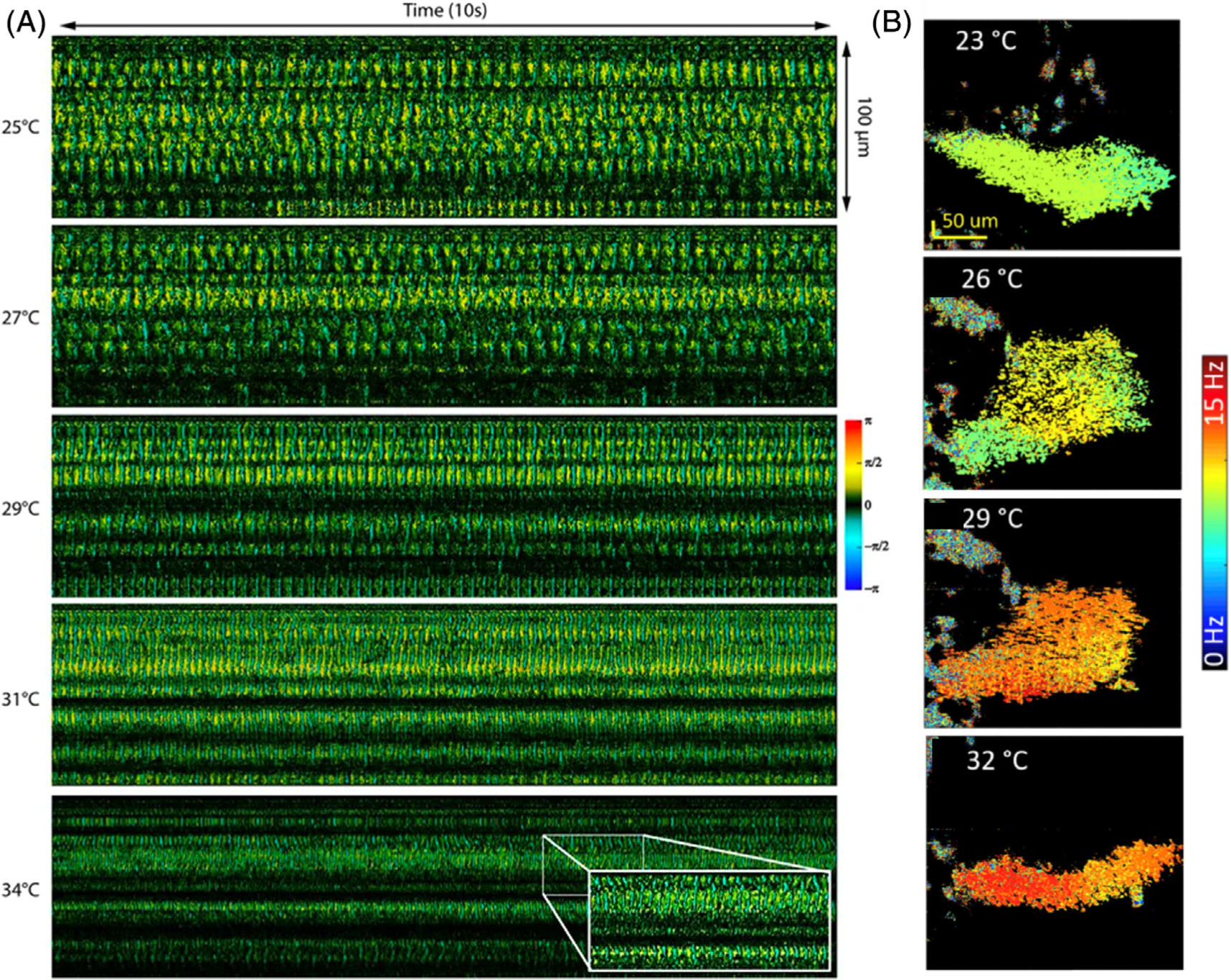
(A) Doppler images of ciliary motion at temperatures of 25°C, 27°C, 29°C, 31°C, and 34°C, in which cilia beating frequency under different temperatures were observed. (B) Spatial distribution of the cilia beating frequency at 23°C, 26°C, 29°C, and 33°C in which temperature has a positive impact on ciliary activity. Adapted from References [86, 87]

Ciliary activity, characterized by the synchronized beating of ciliary cells, generates the primary driving force for mucosa transportation. The dysfunction of ciliary motion could lead to a number of severe diseases, including respiratory disorders and infertility. Doppler OCT has the capability of providing a noninvasive and high-sensitivity imaging tool for evaluation of cilia motion. Jing et al. developed a high-speed Doppler OCT system to visualize temporal cilia beating for studying the influence of external factors on cilia beating frequency, including temperature and albuterol, as shown in Figure 10A [86]. Recently, He et al reported phase-resolved Doppler spectrally encoded interferometric microscopy for real-time visualization of surface dynamics of the oviduct to characterize the ciliary beating frequency in the oviduct, as shown in Figure 10B [87].

### 4.5 | Optical coherence elastography

In addition to angiography and flowmetry, Doppler OCT has also been extended to the application of elastography. OCE has the same resolution as OCT, and its superior displacement sensitivity and high imaging speed make phase-resolved OCE a prominent technique for elasticity measurements. In OCE, an external or internal force is applied to induce a localized displacement, which is then detected by OCT. With its high sensitivity, phase-resolved Doppler OCT measures the phase change which is converted to relative displacement using Equation (8):

(8) $$\Delta d=\frac{\lambda }{4\pi n}\Delta \varphi$$

where *n* is the tissue refractive index, and *λ* is the central wavelength of the light. The absolute displacement can be obtained by integrating the relative displacement, as shown in Equation (9):

(9) $$d=\int \Delta ddt=\int \frac{\lambda \Delta \varphi }{4\pi n}dt$$

**FIGURE 11:**
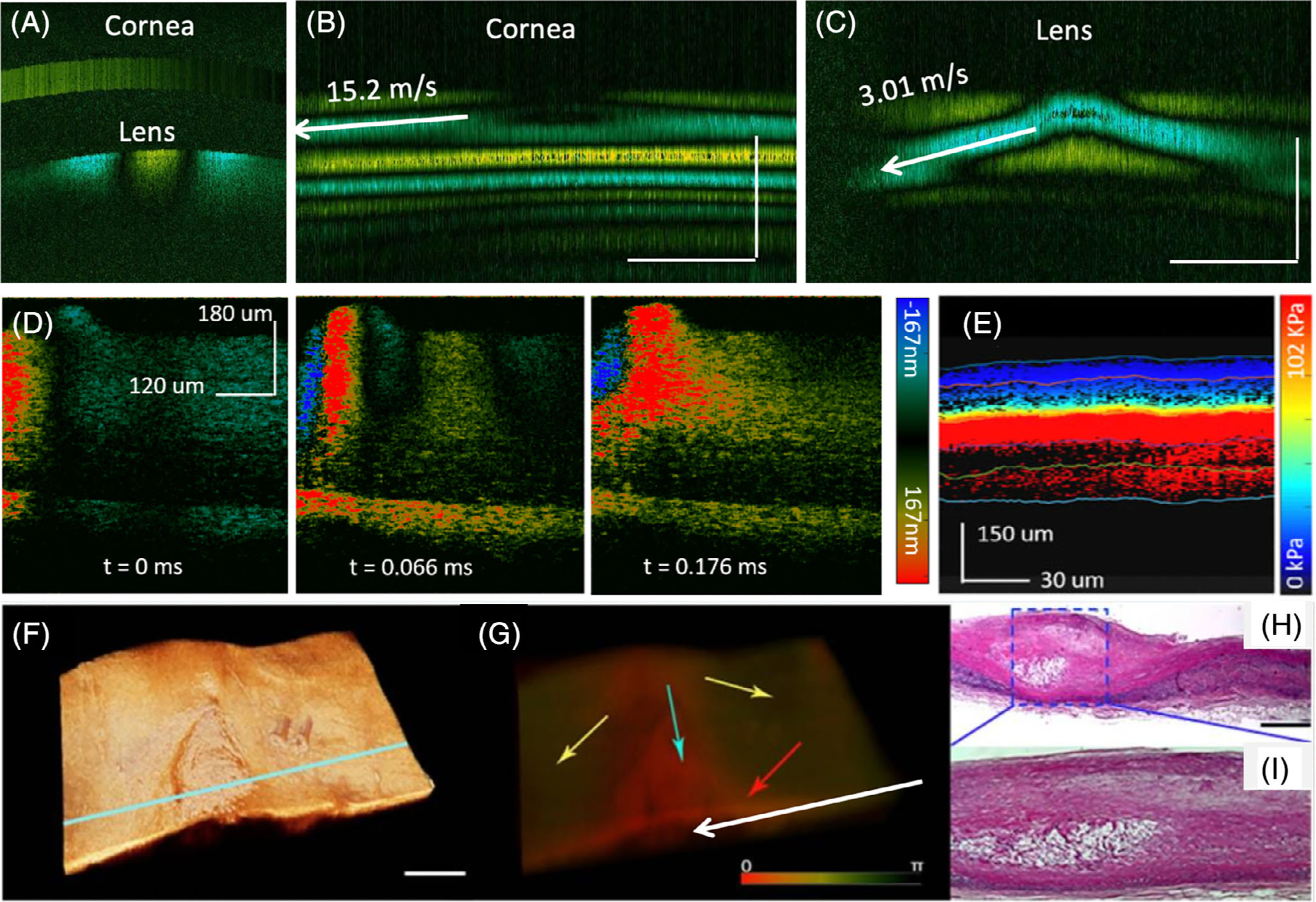
(A) Cross-sectional raw data showing elastic wave propagation of retinal layers at different time points for an ex-vivo pig retina. (B) Elasticity map in rabbit retina in vivo. A different stiffness was demonstrated in different layers of the retina. (C) Doppler OCT image of rabbit cornea and crystalline lens. (D) and (E) Spatiotemporal Doppler OCT images of cornea and lens, respectively. (F) OCT structural and (G) Doppler OCT images of a human cadaver coronary artery. (H) Histological image and (d) close-up view of an atherosclerotic lesion. The red-colored region denoted by the blue arrow in (I) exhibits smaller phase and displacement and, therefore, indicates less elastic, stiffer tissue such as plaques. Scale bars: 1 mm. Adapted from References [40, 42, 95]

In addition, the intensity and phase variance methods described in Section 2 can also be used to visualize the displacement change, but calibration is required, and the sensitivity is relatively low. Besides displacement measurement, resonance frequency and elastic wave propagation have been proposed to calculate the Young’s modulus [39, 42, 88, 89]. OCE has been widely applied in research to provide quantitative assessment of tissue biomechanical properties [37, 39, 43–47, 49, 88–94]. One of the main applications is in ophthalmology. Stefano et al reported the first in vivo human corneal elasticity with 10 subjects, demonstrating a difference in the induced anterior and posterior stromal displacements. Wu et al reported the ex vivo elasticity measurement of lens and investigated the correlation between lens elasticity and intraocular pressure using OCE. Recently, Li et al developed a swept source-based OCE system that is able to simultaneously assess the elasticities of the crystalline lens and the cornea in vivo (as shown in Figures 11A–C) [95]. For elasticity measurement of posterior eye, Qu et al reported the first in vivo quantitative elasticity map of the retina by displacement measurement (ie, the compression approach) in which a difference was demonstrated between healthy and damaged rabbit retina in 2018 [45]. In addition, He et al presented a quantitative method of mapping the mechanical elasticity of the posterior eye based on shear wave method in 2019 in which the elasticity of different layers of retina were quantified, as shown in Figures 11D, E. In addition to ophthalmology, OCT has been applied in cardiology. In 2012, Qi et al demonstrated the first OCE for quantification of plaque by a microscopic system, as shown in Figures 11F–I [40]. Qu et al reported an intravascular endoscopic OCE system in which a miniature focused ring transducer was assembled into an imaging probe to provide ultrasound excitation to detect atherosclerosis plaques [48]. The performance of the system and probe were validated using cadaver tissue. Furthermore, OCE has also been applied in blood coagulation, breast cancer, skin pathology and airway compliance for elasticity quantification as well as mechanobiology research to study the mechanical responses of microparticles [96–101]. Figure 11 shows representative OCE images from different applications.

## 5 | LIMITATIONS AND FUTURE DIRECTIONS OF DOPPLER OCT AND OCTA

Phase-resolved Doppler OCT requires measurement of angles between the OCT detection beam and the blood vessels to quantify the flow velocity. Although quantification of the angle over large numbers of vessels is computer-intensive, Qi et al have demonstrated a volumetric vessel reconstruction approach which enable calculation of Doppler angles to determine the absolute blood flow velocity over a large field-of-view [102]. Alternatively, these problems can be solved by employing angle-independent imaging methods, such as multiple OCT detection beams [103, 104] or synthetic subaperture [105] in which several Doppler angles are utilized to extract velocity components to calculate the absolute velocity. In angiography applications, such as the algorithms summarized in Table 3, the absolute velocity can be determined through pre-calibration (ie, experimentally defining the correlation between SD vs flow velocity); nonetheless, this can only be used for flow velocity within the dynamic range as faster flow can cause signal saturation [30].

The dynamic range of Doppler OCT is confined by phase wrapping, as the phase shift is mathematically restricted to [−π, +π], limiting the ability to detect higher flow speed that is outside of the dynamic range. This issue can be addressed by increasing the Doppler angle or scanning speed or varying the time interval. However, these may degrade the image quality as well as increase the acquisition time. Recently, an automated phase unwrapping algorithm [106] has been proposed in which the magnitude of the phase shift gradient is calculated to correct the wrapping. In addition, phase wrapping correction and discontinuity improvement have also been demonstrated using a two-dimensional unwrapping method [9]. In 2018, Wei et al reported a novel scanning pattern for achieving high dynamic range in which the improved flow dynamic range can be achieved by generating three B-scans of different time intervals [107].

As Doppler OCT ultimately relies on determining the temporal phase shift of the interference signal, the phase stability of the imaging system is a critical key factor in obtaining accurate measurements. Spectral domain OCT (SD-OCT) is commonly considered as the optimal method to achieve high phase stability because of the static operation principle utilized by its spectrometer. While SD-OCT can provide high-precision phase measurements, it has the inherent disadvantage of phase washouts [108]. On the contrary, swept source OCT (SS-OCT) for phase measurements are more widely used as it can provide a higher imaging speed than SD-OCT. Although the operation of SS-OCT has less phase stability, several techniques have been reported to resolve this issue, including optimizing synchronization through the use of a lambda (wavelength) trigger and/or signal timing delay [109], and utilizing a common path setup [110].

In order to perform the Doppler algorithm, multiple temporal data of either an A-line or a B-frame of the same location is required. In OCT, this is typically achieved via a scan apparatus, whose scan speed depends on the light source of SS-OCT and camera speed of SD-OCT. Because the blood flow is relatively slower than the physiological bulk motion, acquiring the temporal data often also captures motion-based artifacts. These artifacts can be corrected by using histogram-based methods to extract and remove the phase change induced by bulk motions [20]. A scanning protocol has been proposed to remove the bulk motion from periodic physiological bulk motion in which a stitch scan protocol in the slow scan direction is applied to stagger bulk motion [111]. Volumetric averaging can also be applied to remove the bulk-motion artifacts, but it greatly increases the imaging time as several volumetric datasets are required. Furthermore, a motion-tracking sensor also can be a possible solution to remove the bulk motion [112].

In Doppler-based OCTA, vascular permeability or leakage cannot be easily visualized due to the lack of image contrast as the amplitude/phase fluctuations of the interference signal are minimal in blood vessels with quasi-static blood flow. Recently, Winkelmann et al have reported a spectral contrast technique for OCTA in which spectral signatures of blood in the visible range are applied to achieve angiograms without the need of blood movement or temporal information [113]. In addition, the motion artifacts can be eliminated as only a single scan is required, but this method cannot quantify flow rates and also has limited penetration depth.

Additionally, reconstruction of smaller vessels and capillaries is often challenging in OCTA as the induced signal fluctuations are more marginal. Several processing algorithms have been used to increase the SNR for imaging capillaries, chiefly by improving the imaging contrast or solving for blood vessel discontinuity [27, 114]. Hessian-Frangi filter is one of the most commonly used techniques in OCTA for improving the visualization of discontinuous vasculature [115]. Tan et al demonstrated a modified Bayesian residual transform-based processing algorithm to reduce speckle noise and motion-related artifacts [114]. Recently, Lee et al incorporated artificial intelligence into OCTA and demonstrated increased detail of the superficial retinal vasculature [77].

Since OCT is an optical imaging technique that mostly relies on light in the near-infrared spectrum, it has a shallow penetration depth (1–2 mm), which limits the utility of OCTA to only the superficial vasculature. Recently, Li et al constructed an SS-OCT system for intravascular imaging using a broadband laser with a center wavelength of 1.7 μm, demonstrating an extended penetration depth compared to conventional OCT systems utilizing shorter wavelength light sources [116]. Dual-axis OCT has also been proposed to improve penetration depth by Zhao et al [117]. In addition to hardware improvements, algorithms have been incorporated to extend the imaging depth. For instance, the scattering reflection matrix approach has been proposed to address the issue caused by multiple scattering although real-time imaging remains a challenge due to the required long acquisition time [118]. Lastly, multimodal imaging systems that incorporate OCT with ultrasound and/or photoacoustic to provide complementary information have also been reported [119, 120].

Several of the aforementioned limitations, including dynamic range, signal saturation and motion artifacts, can be improved by increasing imaging speed of the OCT system; overcoming these limitations will further facilitate the clinical translation of Doppler OCT techniques. Currently, a Fourier-domain mode-locking (FDML) laser with an A-line scan rate in the MHz range has been commercially available, enabling high-speed volumetric scanning for OCTA. An imaging speed of up to 4.7 volumes/s has been demonstrated using FDML, along with improved image contrast [121, 122]. Lee et al. have also developed full-field OCT that applies parallel illumination to achieve high-speed *en face* imaging [123]. Data acquisition and processing speed can be further improved through both hardware and software optimizations, including using high-bandwidth digitizers and utilizing a parallel processing scheme.

Currently, quantitative data analysis in both OCT and OCTA are computationally intensive and less efficient. The accuracy may suffer because of the large volume of data generated by high speed imaging systems. Machine learning has been used in segmentation of OCT structure as well as OCTA. The applications of artificial intelligence in OCT and OCTA, although still at an early stage, have great potential to increase the accuracy and efficiency of quantitative analysis [73, 77, 124].

## 6 | SUMMARY

Doppler OCT as a foundational basis of functional imaging provides noninvasive techniques for quantitative and dynamic evaluation of numerous tissue physiology and pathophysiology in vivo. In angiography and blood flowmetry, different combinations of the processing algorithm, averaging method, and scanning protocol are designed for specific applications, enabling detection and characterization of a broad spectrum of diseases. The best experimental results are often obtained by identifying the optimal balance between the acquisition time, imaging depth and field of view and system cost. In summary, functional extensions of OCT based on the Doppler principle reveal additional tissue characteristics that are not available through conventional OCT, and the reported literature as well as the current state of research have demonstrated Doppler OCT and OCTA as a promising clinical tool in vasculature visualization, flow velocity quantification, and elasticity measurement. Although Doppler OCT and OCTA have been widely applied in ophthalmology, a large number of clinical applications of this technology remains to be explored.

## Figures and Tables

**TABLE 1:** Summary of current angiography methods

Performance method	Lateral resolution	Axial resolution	Flow velocity sensitivity	Invasiveness
ICG angiogram [52, 53]	●●	None	None	Yes
Laser Doppler flowmetry [54, 55]	●●	None	●	None
Doppler ultrasound [56]	●	●	●●	None
Laser speckle [57, 58]	●●	None	●	None
Doppler OCT [6–8, 10, 20–33].	●●	●●	●●	None

*Note*: ●●●, excellent; ●●, good; ●, moderate.

**TABLE 2:** Summary of current algorithms of optical coherence tomography angiography

Method	Algorithm	
Doppler variance	Intensity-based [7]	$\sigma^{2}=1-\frac{2\times \sum_{m=1}^{M-1}\left(\left|F_{m}\right|\times \left|F_{m+1}\right|\right)}{\sum_{m=1}^{M-1}{\left|F_{m}\right|}^{2}+\sum_{m=1}^{M-1}{\left|F_{m+1}\right|}^{2}}$
	Phase-resolved [6, 18]	$\sigma^{2}=1-\frac{2\times \left|\sum_{m=1}^{M-1}\left(F_{m}\times F_{m+1}^{*}\right)\right|}{\sum_{m=1}^{M-1}{\left|F_{m}\right|}^{2}+\sum_{m=1}^{M-1}{\left|F_{m+1}\right|}^{2}}$
Amplitude decorrelation [21]	$\sigma^{2}=1-\frac{1}{M-1}\times \sum_{m=1}^{M-1}\frac{2\times \left|F_{m}\right|\times \left|F_{m+1}\right|}{{\left|F_{m}\right|}^{2}+{\left|F_{m+1}\right|}^{2}}$	
Speckle variance [29]	$\sigma^{2}=\frac{1}{M}\times \sum_{m=1}^{M-1}{\left(\left|F_{m}\right|-\frac{1}{M}\sum_{m=1}^{M}\left|F_{m}\right|\right)}^{2}$	
SD [30]	$R=\frac{\sqrt{\frac{1}{M-1}\times \sum_{m=1}^{M-1}{\left(\left|F_{m}\right|-\frac{1}{M}\sum_{m=1}^{M}\left|F_{m}\right|\right)}^{2}}}{\frac{1}{M}\sum_{m=1}^{M-1}\left|F_{m}\right|}$	
Differentiation	Intensity-based [22]	$I=\frac{1}{M-1}\times \sum_{m=1}^{M-1}\|F_{m}\left|-\right|F_{m+1}\|$
	Intensity and phase-based [31]	$I=\frac{1}{M-1}\times \sum_{m=1}^{M-1}\left|F_{m}-F_{m+1}\right|$
Phase variance [32, 33]	$PV_{v}=\frac{1}{M-1}\sum_{m=1}^{M-1}{\left(\Delta \varphi_{m}-\frac{1}{M-1}\sum_{m=1}^{M}\Delta \varphi_{m}\right)}^{2}$	
	$\Delta \varphi_{m}=\varphi_{m+1}-\varphi_{m}$	

*F*_*m*_ and *F*_*m* + 1_: OCT complex data from the same location but at different time points. *M*: time repeated at one location. Δφ: phase change. *σ*^2^: variance. *R*: SD. I: intensity of OCT signal.

**TABLE 3:** Summary of scanning protocols

	Inter-A-lines	Inter-frames
Principle	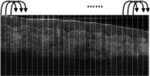	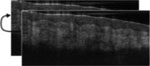
Advantage	Less motion artifact	High sensitivity for slow velocity
Disadvantage	Low sensitivity for slow velocity	More motion artifact

**TABLE 4:** Summary of current average methods

		Principle	Advantage	Disadvantage
Averaging method	Split spectrum	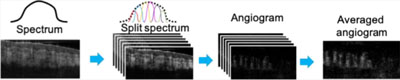	High speed	Low axial resolution
	Volumetric	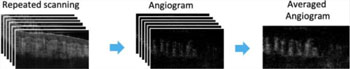	High sensitivity	Long acquisition time
